# Sampling and metabarcoding of arthropod environmental DNA traces from flowers

**DOI:** 10.1016/j.mex.2025.103329

**Published:** 2025-04-23

**Authors:** David Wari, Yoshinobu Kusumoto, Toshio Kitamura

**Affiliations:** a Western Region Agricultural Research Center (WARC), National Agriculture and Food Research Organization (NARO), WARC Main Campus, Fukuyama, Hiroshima, Japan; b Western Region Agricultural Research Center (WARC), National Agriculture and Food Research Organization (NARO), Shikoku Research Station, Zentsuji, Kagawa, Japan

**Keywords:** Arthropods, Environmental DNA (eDNA), Flowers, Insectary plants, Metabarcoding, Natural enemies, Next Generation Sequencing (NGS), Method for detecting arthropod environmental DNA traces on flowers

## Abstract

Screening and selection of insectary plants that promote natural enemies has been mostly approached via conventional methods that employ mundane man-hours of manual surveys, sampling, sorting, and viewing under microscope. In this digital age, mundane man-hours in ecological surveys can be approached with revolutionary sequencing technology, i.e. the Next Generation Sequencing (NGS). Ecological scientist, especially marine biologists have been tracing environmental DNA (eDNA) by utilizing the NGS technology to study/monitor micro- and macro-organisms in aquatic conditions. The eDNA technology has now been adopted by applied entomologists and ecologists to survey and monitor arthropod biodiversity through space and time. Several advancements have been made in detecting arthropod eDNA traces cryopreserved in plant tissues such as stems, branches, leaves, and flowers. Using the techniques developed thus far, we adopted, and slightly modified the method originally intended for aquatic studies to evaluate arthropod eDNA traces on flowers of flowering plants. Corroborating the method, we showed that eDNA traces washed off from floral parts revealed plethora of arthropod species. Furthermore, data sets generated from eDNA analysis may assist in improving the data gathered using conventional methods.

• Conventional methods used in surveying arthropod fauna (especially indigenous natural enemies) on flowering plants can be a tedious exercise. Here, we adopted and optimized the method initially designed for aquatic environmental DNA analysis to supplement conventional methods in arthropod studies.
• The optimized method showed that traces of arthropod eDNA materials on floral parts are detectable.
• The eDNA technology can be used to generate qualitative data, especially data on the cryptic and unknown species of flowering plant-associated-natural enemies, that can supplement the quantitative data gathered from conventional methods.

## Graphical abstract

Schematic representation of the environmental DNA sampling from flower heads through to metabarcoding.

**Figure:**
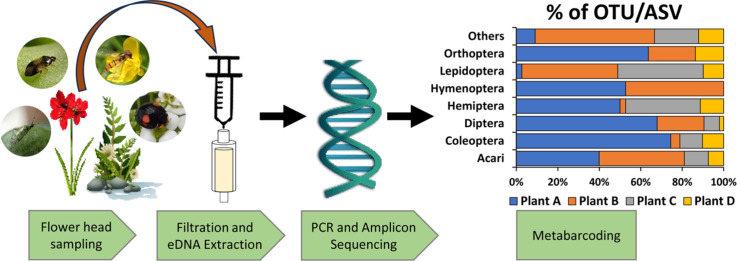

Specifications table

**Table:** 

Subject area:	Agricultural and Biological Sciences
More specific subject area:	Metabarcoding flower-arthropod biodiversity
Name of your method:	Method for detecting arthropod environmental DNA traces on flowers
Name and reference of original method:	M. Kawato, T. Yoshida, M. Miya, S. Tsuchida, Y. Nagano, M. Nomura, A. Yabuki, Y. Fujiwara, K. Fujikura. Optimization of environmental DNA extraction and amplification methods for metabarcoding of deep-sea fish. MethodsX 8 (2021) 101,238. (https://doi.org/10.1016/j.mex.2021.101238)
Resource availability:	Information regarding the resources required to reproduce the results acquired in this study are all affixed in this article.

## Background

In recent years, environmental DNA (eDNA) metabarcoding approach has demonstrated the possibility of obtaining information to assist in monitoring micro- and macro-organisms in both aquatic and terrestrial communities [1]. Hitherto, tremendous progress has been made on how to sample traces of eDNA from different environmental conditions, sampling of eDNA materials [2,3,4], eDNA extraction protocols [5], primer designs [6,7] and their applicability to a variety of biological communities. Furthermore, a variety of PCR kits used in eDNA amplifications and data analysis of biological communities in aquatic [4,5,8], terrestrial [3,9,10] and atmospheric [11] conditions are but few examples to name. In terrestrial environments relating to pest management, the eDNA technology could be significant help. First, the advancement of eDNA technology to cover a variety of fields could add some ease to the ecological surveys that employ conventional methods with mundane man-hours of sampling, sorting and screening. Next, the eDNA technology could also aid in providing an alternate perspective of information that cannot be manually obtained through conventional methods. Last but not the least, the eDNA technology could minimize the time needed to sample, sort and screen for the targeted biological communities. With these parameters in mind, combined with the plethora of information on the eDNA technology on hand, we adopted and optimized the eDNA sampling and metabarcoding technique to survey arthropod fauna inhabiting (or visiting) the insectary flowering plants.

Screening insectary flowering plants to be used in augmenting indigenous natural enemies is an important task in Conservation Biological Control (CBC). Given that some indigenous natural enemies are laborious to observe using conventional methods, metabarcoding techniques that can track or monitor indigenous natural enemy eDNA traces would be of significant help. As such, we adopted and modified the sampling and metabarcoding method used in surveying and monitoring aquatic biological communities by Kawato et all [5] to survey the overall arthropod communities (especially indigenous natural enemies) inhabiting insectary flowering plants. Preliminary data showed in this article confirms that the adopted and optimized eDNA method can detect traces of arthropod species from both herbivores and their indigenous natural enemies on host flowering plants. This eDNA method, therefore, can be used in screening of flowering plants as potential insectary plants that hosts indigenous natural enemies. Furthermore, techniques developed in this study (eDNA technology) can also be applied in both basic and applied research for designing pest management strategies.

## Method details

### Materials (equipment, kits and reagents) required for sampling and extraction of eDNA in this protocol

I. A pair of clean latex surgical gloves
II. 500 ml beaker and/or bottle
III. Clean/sterilized tweezers
IV. Clean/sterilized scissors
V. Sterilized lancet or surgical blade
VI. Sterilized 2.0 ml and 1.5 ml tubes
VII. PVC pipe cutter (Konyo, Japan)
VIII. 60 ml syringe with a luer lock (Takefu Original Production, Japan)
IX. Sterivex cartridge (Merck Millipore, Germany)
X. RNA*later* (ThermoFisher, USA)
XI. PBS buffer (Nippon Gene, Japan)
XII. DNeasy Blood and Tissue Kit (Qiagen, USA)

### Sampling and filtration of arthropod eDNA

1. After sampling the flower heads, brush off as much as possible, any live specimens of arthropods on the floral parts.
2. Place the sampled flower heads (number of flower heads depends on the flower species, 4 to 10 flower heads were enough to filter arthropod eDNA traces in this experiment) into a 500 ml bottle containing sterilized water and cap tightly.
3. Agitate the bottle briefly and transfer the fluid into a clean 500 ml beaker.
4. Using a 60 ml syringe with luer and a Sterivex cartridge, filter the fluid.
5. Add 1.0 ml RNA*later*, label the sample and store in −15 to −20 °C freezer until eDNA extraction.
6. Optionally, proceed onto eDNA extraction (step 9) without adding RNA*later* if extracting eDNA on the same day as sampling.
7. Remove the Sterivex cartridge from the freezer and thaw on either a work bench or a clean bench at room temperature. To minimize contamination, working in a clean bench is highly recommended.
8. After thawing, aspirate RNA*later* from the Strivex cartridge using a syringe or a vacuum pump, whichever that befits the laboratory conditions.
9. Place the Sterivex cartridge onto a sterile petri dish. From this step onwards, to minimize contamination, use a pair of sterilized latex surgical gloves and perform all necessary procedures in a clean bench. Additionally, if working multiple samples, make sure to sterilize the tweezers, scissors, lancet and PVC pipe cutters.
10. Using a PVC pipe cutter, cut the Sterivex cartridge from the inlet connection edge of the cartridge (NOT the outlet edge), separate the housing unit from the filter unit using a sterilized tweezer, and place the filter unit on a separate sterile petri dish.
11. Using a sterilized lancet, carefully lacerate the filter paper around the edges at both ends of the filter unit as well as along the filter unit then detach the membrane from its housing/holder. Be careful not to let the filter membrane dry out.
12. Quickly but surgically incise the filter membrane into 26 to 32 micro-fragments using a pair of sterilized scissors. Since the micro-fragments are now very small in size, chances are that they might dry up in seconds. Caution must be maintained to keep the micro-fragments wet for a quality eDNA yield.

### eDNA extraction, adopted from Kawato et al. [5] and DNeasy blood and tissue kit with slight modifications

The eDNA extraction method is adopted and slightly modified from Kawato et al. [5] and the Qiagen DNeasy blood and tissue kit that include steps for lysis, binding, first and subsequent wash and finally elution. This process may change depending on each laboratory conditions.

1. **[*Lysis*]** Using a sterilized tweezer, retrieve all the micro fragments into a 1.5 ml tube containing the lysis solution of 440 µl PBS buffer, 400 µl Buffer AL, and 40 µl proteinase K (Buffer AL and Proteinase K are supplied together with the DNeasy Blood and Tissue Kit). Close the lid firmly and agitate thoroughly by shaking with hand. At this stage, an incubator or a heat-block dry bath is switched on and preset at 56 °C.
2. Incubate the tubes containing the samples in the lysis solution at a constant temperature of 56 °C for 2 hrs with occasional shaking (at least at an interval of once every 20 min).
3. After the incubation at 56 °C for 2 hrs, centrifuge at 15,000 × *g* for 1 min. While centrifugation, prepare new sterilized 2.0 ml tubes.
4. Carefully transfer the supernatant (approx. 880 µl of the solution) into the new sterilized 2.0 ml tube. Try not to collect debris as much as possible.
5. Add 400 µl of 99.9% ethanol to the supernatant in the 2.0 ml tube and mix thoroughly by pipetting.
6. Prepare DNeasy spin columns (supplied together with the DNeasy Blood and Tissue Kit) by placing them in a collection tube (supplied together with the DNeasy Blood and Tissue Kit).
7. **[*Binding*]** Since the DNeasy mini spin column can only be filled up to 700 µl, transfer up to 700 µl of the mixture in Step 5 into the DNeasy mini spin column. Then centrifuge at 6000 × *g* for 1 min.
8. Discard the flow-through that’s collected in the collection tube and place the DNeasy mini spin column into a new collection tube. Add the remaining solution and centrifuge again at 6000 × *g* for 1 min. Discard the collection tube and place the column into a new collection tube.
9. **[*First wash*]** Add 500 µl of Buffer AW1 (supplied together with the DNeasy Blood and Tissue Kit) to the column and centrifuge at 6000 × *g* for 1 min. Discard the collection tube and place the column in a new collection tube.
10. **[*Subsequent wash*]** Add 500 µl of Buffer AW2 (supplied together with the DNeasy Blood and Tissue Kit) to the column and centrifuge at 20,000 × *g* for 3 min.
11. Discard the flow-through, place the column back in the empty collection tube. To completely dry the column from residual ethanol from buffer AW1 and AW2 that may interfere with subsequent steps, an additional centrifugation at 20,000 × *g* for 1 min is needed.
12. Discard the collection tube and place the column in a new sterile 1.5 ml tube.
13. **[*Elution*]** Add 200 µl Buffer AE (supplied together with the DNeasy Blood and Tissue Kit) directly onto the DNeasy membrane in the column then incubate at room temperature (15–25 °C) for at least 1 min. After incubation, centrifuge at 6000 × *g* for 1 min.
14. Discard the column and cap tightly the tube containing the eluted eDNA solution, label and store in a freezer (−15 to −20 °C) till further experiments.

### PCR amplification (first PCR)

First PCR was performed using the KAPA HiFi HotStart ReadyMix DNA polymerase (KAPA Biosystems, USA). Primer sets of 1st-gInsect-F (5′ ACACTCTTTCCCTACACGACGCTCTTCCGATCT-NNNNNNGATAGAAACCAACCTGGCT 3′) and 1st-gInsect-R (5′ GTGACTGGAGTTCAGACGTGTGCTCTTCCGATCT-NNNNNNGACGAGAAGACCCTATA 3′) targeting the 16S rRNA region were supplied by the Bioengineering Lab. Co., Ltd (Sagamihara, Japan) and used for the first PCR. The composition of the PCR mixture followed recommendations by the KAPA Biosystems with slight modifications. PCR reaction was performed in a 25 µl total volume mixture containing KAPA HiFi HotStart ReadyMix DNA polymerase, 10 µM each of 1st-gInsect-F and -R primers, 2 µl of the eDNA template and finally measured-up with PCR grade water. PCR conditions were as follows; initial denaturation of 1 cycle at 95 °C for 2 min followed by 35 cycles of denaturation at 98 °C for 20 s, annealing at 60 °C for 30 s and an extension at 72 °C for 30 s, and a subsequent final extension of 1 cycle at 72 °C for 2 min. After PCR amplification, 3 µl of the PCR product was electrophoresed on a 1% agarose gel (Nippon Gene, Japan) in TAE (40 mM Tris, 40 mM acetic acid, and 1 mM ethylenediaminetetraacetic acid) stained with SYBR Safe DNA gel stain (Invitrogen, USA) and observed under WSE-5400-UP Printgraph Classic (ATTO Corporation, Japan) mounted on a Safe Imager Blue Light Transilluminator (Invitrogen) to confirm if the eDNA extraction was a success. The remaining 22 µl of the first PCR product was then sent to the Bioengineering Lab. Co., Ltd for library construction, amplicon sequencing and analysis.

Optionally, KAPA2G Robust HotStart ReadyMix DNA polymerase (KAPA Biosystems) can also be used for DNA check. PCR mixture and PCR conditions are slightly different from the KAPA HiFi HotStart ReadyMix DNA polymerase. The PCR mixture composition can be performed in a 10 µl total volume mixture containing KAPA Robust HotStart ReadyMix DNA polymerase, 10 µM each of 1st-gInsect-F and -R primers, 2 µl of the eDNA template and measured-up with PCR grade water. PCR conditions are as follows; initial denaturation of 1 cycle at 95 °C for 3 min followed by 30 cycles of denaturation at 95 °C for 15 s, annealing at 60 °C for 30 s and an extension at 72 °C for 30 s, and a subsequent final extension of 1 cycle at 72 °C for 2 min. PRC products can be size-fractionated on a 1% agarose gels and visualized under UV light.

### Library construction, amplicon sequencing and data analysis

Library construction, amplicon sequencing and data analysis from the first PCR were performed by Bioengineering Lab. Co., Ltd. In brief, after purification of the first PCR products using VAHTS DNA Clean Beads (Vazyme Biotech Co., Ltd., China), the concentrations of the purified first PCR product was measured using Synergy H1 (Agilent Technologies, USA) and QuantiFluor dsDNA System (Promega, USA). To construct the library, second PCR was performed using second PCR primers with appropriate unique index sequences. The PCR reaction was performed in a 10 µl mixture containing 2x PCR buffer for KOD FX Neo (TOYOBA, Japan), 2 mM each of dNTPs, 5 µM each of 2nd PCR primers; 2nd-F (5′ ATGATACGGCGACCACCGAGATCTACAC-Index2-ACACTCTTTCCCTACACGACGC 3′) and 2nd-R (5′ CAAGCAGAAGACGGCATACGAGAT-Index1-GTGACTGGAGTTCAGACGTGTG 3′), 1 µl of the purified first PCR product, 1.0 U/µl of KOD FX (TOYOBA) and measured-up with nuclease-free water. PCR conditions were as follows; initial denaturation of 1 cycle at 94 °C for 2 min followed by 10 cycles of denaturation at 98 °C for 10 s, annealing at 60 °C for 30 s and an extension at 68 °C for 30 s, and a subsequent final extension of 1 cycle at 68 °C for 2 min. After the 2nd PCR, the PCR product were purified using the VAHTS DNA Clean Beads. The concentration of the libraries were measured using Synergy H1 and QuantiFluor dsDNA System followed by library quality check confirmation using Fragment Analyzer and dsDNA 915 Reagent Kit (Agilent Technologies). Libraries were sequenced using MiSeq system and MiSeq Reagent Kit v3 (Illumina, San Diego, CA, USA) under 2 × 300 bp conditions.

The resulting paired-end illumina sequence reads were processed and analyzed in QIIME 1 (http://qiime.org/index-qiime1.html) and QIIME 2 pipelines (https://qiime2.org/). For QIIME1 analysis, paired-end sequence reads matching the primer sequences were extracted using fastx_barcode_splitter tool of FASTX-Toolkit (ver. 0.0.14). Primer sequences were removed using fastx_trimmer of the FASTX-Toolkit followed by removal of unreliable sequence reads using sickle (ver. 1.33). Paired-end sequence reads were then assembled using FLASH (ver. 1.2.11) under the conditions of combined sequence length of 470 bases, read length of 280 bases, and minimum overlap length of 10 bases. Operational Taxonomic Units (OTUs) table was then created using usearch (ver. 11.0.667) under the condition of 97% sequence homology. Finally, BLASTN (ver. 2.13.0) search was performed for the representative paired-end sequences of the OTUs. As for QIIME 2, first, the sequence reads matching the primer sequences were extracted using the fastx_barcode_splitter tool of FASTX-Toolkit (ver. 0.0.14). Primer sequences and unreliable sequence reads were removed using fastx_trimmer of the FASTX-Toolkit. Paired-end sequence reads were then assembled using the FLASH (ver. 1.2.11). After quality check and removal of chimera sequences and/or noise sequences using QIIME 2 (ver. 2023.7) dada2 plugin, representative paired-end sequence reads were tabulated into Amplicon Sequencing Variants (ASVs). The representative ASVs were then blast searched in the NCBI BLAST Nucleotide Database (nt) (ver. 2.13.0). Other parameters were maintained under standard conditions.

## Method validation

### Electrophoresis after first PCR

**Fig. 1:**
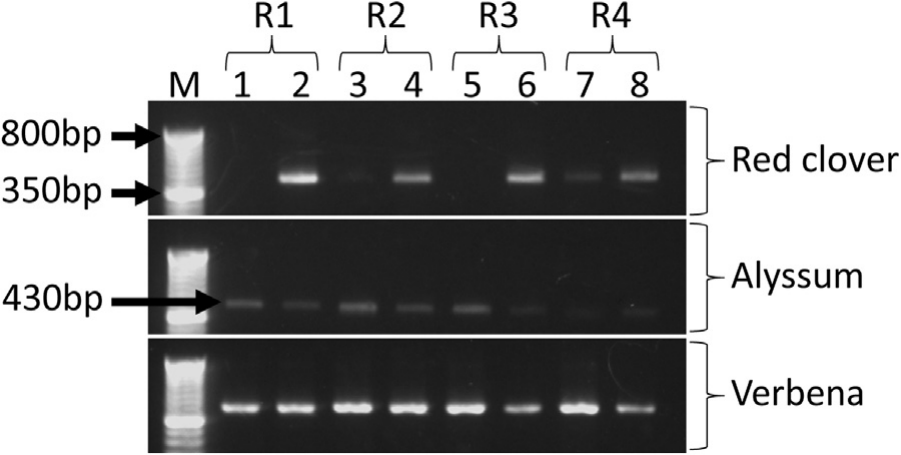
Agarose gel electrophoresis image of arthropod eDNA traces from Red clover, Alyssum and Verbena flower heads amplified using the 1st PCR gInsect primers. Four replicates from each of the flower species (R1, R2, R3 and R4) were made. For each replicate, the flowers were washed twice and filtered into two different Sterivex cartridges. Numbers in the Odd numbered lanes (i.e. 1, 3, 5 and 7) indicates the first wash, while the Even numbered lanes (i.e. 2, 4, 6 and 8) indicate the second wash. M indicates the Invitrogen 50 bp DNA ladder used in this study.

To sample arthropod eDNA traces from flower tissues, the flower heads were placed in a 500 ml bottle containing 500 ml of sterilized water, capped tightly and briefly agitated. The fluid was emptied into a clean beaker and filtered using a syringe and a Sterivex cartridge. This was treated as the first wash. The emptied bottle containing the flower heads were then filled with sterile water (500 ml) and repeated the process as discussed in the first wash. This was treated as the second wash. Arthropod eDNA traces trapped in the 0.45 µm membrane were then extracted using the Qiagen blood and tissue kit following the manufacturer’s instructions with slight modifications. The resultant eDNA were then amplified using the 1st PCR gInsect primers and the PRC products size-fractionated on 1 % agarose gels and visualized under UV light as discussed above. The results of the gel electrophoresis are shown in Fig 1. Arthropod eDNA traces were successfully extracted from flowers using the optimized method used in tracing eDNA from aquatic conditions. As shown in Fig. 1, while eDNA was successfully extracted in the first and second wash from alyssum and verbena flower heads, two samples from red clover in the first wash, however, failed to amplify. There are various reasons why no amplification was observed in the second wash for red clover. Nonetheless, one possible reason could be due to the cuticle wax on the flower surfaces. Cuticle wax is the main interface between the plant tissue surface and its surrounding environment with the sole function of protecting the plants integrity from biotic and abiotic stressors [12]. The cuticle wax are lipids made of hydrophobic (water-hating) natural components such as biopolymer, cutin, and cuticular lipids [12,13]. It’s a possibility that the hydrophobic lipids on flower surface could play a role in repelling water while still confining the arthropod eDNA traces on flower surfaces. The second wash somehow destabilize the cuticle wax resulting in extraction of arthropod eDNA traces in the second wash. While this is only a hypothesis, further studies are needed to confirm this conjecture. Floral structure such as that of the trichomes cannot be ruled out in trapping arthropod eDNA materials. Nevertheless, multiple washes (2 or more washes) is highly recommended to destabilize any hydrophobic compounds on floral surfaces from interfering with the sampling and extraction process. Furthermore, the results shown here are indicative that arthropod eDNA traces washed off from the floral heads in a 500 ml of sterilized water (half that of the usual amount i.e. 1 L as reported in aquatic biological community studies [2,5,14]) is plausible.

### Detection of environmental DNA traces of arthropod communities

**Fig. 2:**
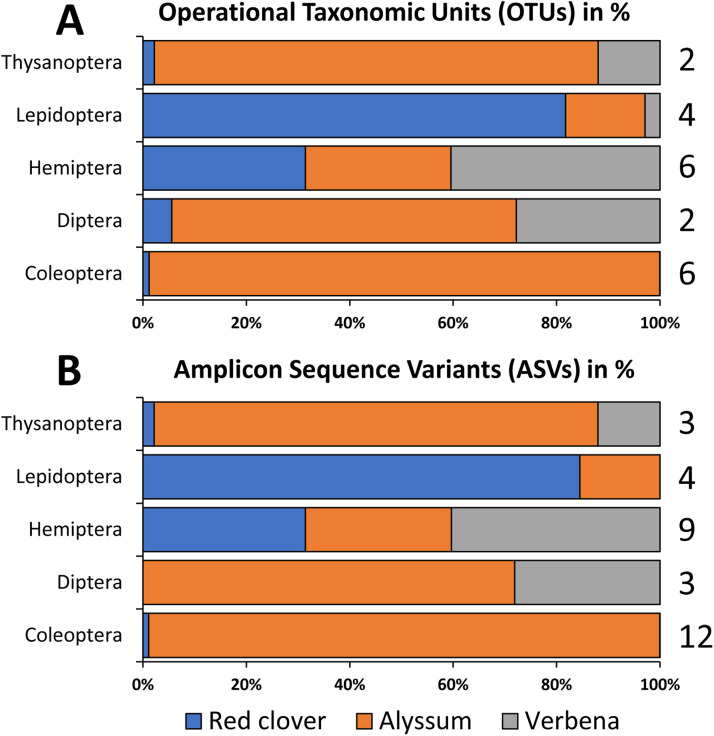
Arthropod eDNA traces from Red clover, Alyssum and Verbena. The bar plots shows the per cent composition of OTUs (A) and ASVs (B) by Arthropod order (*n* = 4). Numbers on the right indicate the number of arthropod species recovered in each order.

To detect arthropod communities dwelling on the flowers, the first PCR products from the 1st and 2nd wash (see Fig. 1 for Alyssum and Verbena) were pooled together as one set, while samples that showed only amplification in the 2nd wash (e.g. R1 and R3 samples in Fig. 1 for red clover) were used without pooling the 1st and 2nd washes. The samples (1st PCR product) were sent to the Bioengineering Lab for amplicon sequencing. The results of the amplicon sequencing are presented as OTUs and ASVs in bar plots (*n* = 4) as shown in Fig. 2. While there are no significant differences in the arthropod communities observed between OTUs and ASVs from the two analytical tools (QIIME1 and QIIME2, respectively), the results are evident that arthropod community richness are detectable. In general, both herbivores and natural enemies were detected. As expected, the larger portion of the arthropod eDNA traces were from the herbivores such as the aphids, thrips, moths and beetles from their taxonomic Orders of Hemiptera, Thysanoptera, Lepidoptera and Coleoptera, respectively. Indigenous natural enemies such as *Nesidiocoris tenuis* (Hemiptera), *Orius sauteri* (Hemiptera), *Propylea japonica* (Coleoptera) and *Eupeodes luniger* (Diptera), that predate on aphids and thrips were also detected. These results are consistent with related studies on detecting terrestrial arthropod eDNA traces on flowers [15,16] as well as on whole plants through destructive methods [10] and non-destructive methods [3]. On the other hand, parasitic wasps (important natural enemies of aphids) and pollinators in the taxonomic order ‘Hymenoptera’ were not detected. Whether arthropods in the Order Hymenoptera were not present during the sampling of flower heads or perhaps the primers could not amplify the eDNA traces of parasitic wasps remains to be determined. It must be noted that the flower heads used in this study were sampled from greenhouses in mid-winter to test and validate this method therefore, the data presented in this study does not reflect the abundance and distribution of most arthropod communities that emerges during the warmer seasons. Nonetheless, the method utilized in this study successfully detected arthropod communities from agriculturally important species and as such, we intend to further improve and use this method during our surveys to screen for native plants that hosts indigenous natural enemies.

The continuous development and improvement of NGS technology has been benefiting multitude of research fields [17]. In the entomological fields, especially in pesticide diagnosis, NGS via genotyping-by-sequencing has massively benefited researcher working on pest resistance development and monitoring [18]. Furthermore, NGS has also improved faster elucidation of genomes and large-scale screening of molecular markers for model and non-model organisms [17]. In this digital age, where communities, ideas, procedures, skills and know-hows are evolving in a faster rate, while conventional methods are still a huge part of research procedures, the NGS technology is here to stay. To date, there are multiple studies reporting the use of NGS in metabarcoding terrestrial arthropods on wild flowers [15], detecting pollinators, pests, parasites and beneficial insects on plants [19], observing arthropod interactions on plants [16], monitoring invasive pests [20], detecting cryptic terrestrial arthropods [21], determining spatio-temporal dynamics of arthropod species on plants [10] and the list goes. Overall, optimizing the eDNA metabarcoding technology can be beneficial to a variety of entomological research involving terrestrial arthropod such as surveys of indigenous natural enemies on native plants, supplement conventional methods used in arthropod surveys, raising awareness in arthropod conservation and biodiversity, monitoring of arthropod communities and their diversity inventory, detection of invasive species, plant-pest-natural enemy interactions and designing of pest management programs.

## Limitations and prospects

This method is far from perfect and would need fine tuning to cover multiple aspects of information that’s required in arthropod surveys and monitoring. That’s including careful designing of primers that should amplify natural enemies that occur in extremely minor taxa as well as ones that are nocturnal. The absence of rare and nocturnal species such as predatory phytoseiid mites, parasitoids and green lacewings not detected in this study. Also, the arthropod DNA degradation period on flower heads, i.e., the timeframe at which arthropod deposits their DNA traces until its degradation is still unknown. Whether its the primers thats not amplifying the said arthropod species or the degradation of the eDNA samples needs further analysis. In this study, we used 16S rRNA gene segment to study the arthropod eDNA on flowers. It would be interesting to include the Cytochrome C Oxidase I (COI) gene segment to greatly improve the taxonomic identification of arthropod species. In addition, the chances of potential false positives from a certain insect species could occur given that flowers are exposed to the ecosystem that is inhabited by multitude of arthropod species. Also, the origin of the arthropod DNA could differ when arthropods carry eDNA traces of other arthropod species when moving from one plant to another. Care must be undertaken when deciding on the eDNA reads as once that are expected compared to false positives. Next, does eDNA sequences presented as reads imply species richness or abundance? Studies in aquatic conditions using conventional methods as well as eDNA metabarcoding have shown that species richness and abundance are to some extent comparable [22,23]. In terrestrial conditions, Johnson et al [19] and Stotuth et al [16] studied the population trends of arthropod recovered from eDNA analysis and camera recordings. While the two studies were performed independently from each other, both studies have concluded that eDNA technique was more reliable in detecting arthropod species compared to the camera recordings. To alleviate the short falls of video recordings, other conventional methods such as sticky traps, visual surveys, pitfall traps are suggested. These conventional methods could further aid in determining whether the eDNA metabarcoding can qualitatively and quantitatively determine arthropod richness and abundance on a given flowering plant. Last but not the least, like any other eDNA technology, this method is designed purposely to detect arthropod eDNA traces. When sampling flower heads, different growth stages of arthropod specimens will be present on the flower heads that are invisible to the naked eye which may result in a skewed data. The authors recommend (as mentioned in the methods section) that any small arthropods lacking around should be carefully removed using a brush or something similar. While multitude of arthropod remains such as feces, exoskeletons and body parts, salivars or mouth parts from the arthropod feeding that are left behind on floral parts does fit the classical definition of eDNA, therefore merits their description as eDNAs in the data. However, the possibility that different arthropod growth stages such as eggs or larvae that would somehow be filtered into the sterivex, extracted and analyzed are high. Whether the eDNA from these growth stages such as eggs, first or second instar nymphs/larvae extracted and analyzed in this method be referred to as eDNA traces from arthropods still remains for elaboration. Finally, while NGS provides massive advantages in generating massive data sets, the major bottleneck is the data processing and interpretation. Before data processing, sample biomass, climate conditions and seasons at which samples were collected, organismal decay rates, extraction techniques and kits and many more biotic and abiotic factors will impact the shedding and degradation of the eDNA traces are equally significant in affecting the final eDNA concentration. It is imperative that such conditions are optimized so that reliable data is generated for data interpretation.

## CRediT author statement

**David Wari:** Conceptualization, Methodology, Investigation, Resources, Data Curation, Writing-Original draft preparation; **Yoshinobu Kusumoto:** Conceptualization, Resources, Writing-Review & Editing; **Toshio Kitamura:** Conceptualization, Methodology, Data curation, Supervision, Writing-Review & Editing, Project administration.

## Declaration of competing interest

The authors declare that they have no known competing financial interests or personal relationships that could have appeared to influence the work reported in this paper.

## Data Availability

Data will be made available on request.
